# Electrospun Janus Beads-On-A-String Structures for Different Types of Controlled Release Profiles of Double Drugs

**DOI:** 10.3390/biom11050635

**Published:** 2021-04-25

**Authors:** Ding Li, Menglong Wang, Wen-Liang Song, Deng-Guang Yu, Sim Wan Annie Bligh

**Affiliations:** 1 School of Materials Science and Engineering, University of Shanghai for Science and Technology, Shanghai 200093, China; 182442532@st.usst.edu.cn (D.L.); 191370148@st.usst.edu.cn (M.W.); wenliang@usst.edu.cn (W.-L.S.); 2 Shanghai Engineering Technology Research Center for High-Performance Medical Device Materials, Shanghai 200093, China; 3 School of Health Sciences, Caritas Institute of Higher Education, Hong Kong 999077, China

**Keywords:** side-by-side electrospinning, Janus structure, beads-on-a-string, immediate release, sustained release

## Abstract

A side-by-side electrospinning process characterized by a home-made eccentric spinneret was established to produce the Janus beads-on-a-string products. In this study, ketoprofen (KET) and methylene blue (MB) were used as model drugs, which loaded in Janus beads-on-a-string products, in which polyvinylpyrrolidone K90 (PVP K90) and ethyl cellulose (EC) were exploited as the polymer matrices. From SEM images, distinct nanofibers and microparticles in the Janus beads-on-a-string structures could be observed clearly. X-ray diffraction demonstrated that all crystalline drugs loaded in Janus beads-on-a-string products were transferred into the amorphous state. ATR-FTIR revealed that the components of prepared Janus nanostructures were compatibility. In vitro dissolution tests showed that Janus beads-on-a-string products could provide typical double drugs controlled-release profiles, which provided a faster immediate release of MB and a slower sustained release of KET than the electrospun Janus nanofibers. Drug releases from the Janus beads-on-a-string products were controlled through a combination of erosion mechanism (linear MB-PVP sides) and a typical Fickian diffusion mechanism (bead KET-EC sides). This work developed a brand-new approach for the preparation of the Janus beads-on-a-string nanostructures using side-by-side electrospinning, and also provided a fresh idea for double drugs controlled release and the potential combined therapy.

## 1. Introduction

Nanotechnology has been the most widely studied and multidisciplinary technology field for the past few years. Nanotechnology can control the structure of materials at the nanoscale, so as to obtain significant changes in material properties, outstanding advantages and wide applications. Nanomaterials include nanotubes [1], nanosheets [2], nanorods [3], nanoparticles and nanofibers [4], etc., among which nanofibers have their unique properties to get potential applications in a wide variety of fields [5]. The preparation methods of nanofibers include phase separation, template synthesis, melt spray technology, self-assembly, electrospinning and so on [5,6]. Compared with other preparation methods, electrospinning is a “top-down” electrohydrodynamics process (EHDA, including electrospinning electrospraying and e-jetting) [7,8], with simple operation, better continuity and adjustable functionality. Electrospinning nanofibers have a number of advantages, such as large specific surface area, tremendous porosity, low cost and simple process [9,10].

With the rapid development of preparation and characterization of nanofibers, probable applications of nanofibers have been explored, including biomedicine [11,12,13], food packaging [14,15,16], energy storage and conversion [17,18,19] and environmental applications [20,21,22,23,24]. Nanofibers prepared by electrospinning have been applied in the biomedical field for many attempts, such as drug delivery system [9,25], tissue regeneration of bone [26,27], heart valves [28,29], muscle [30,31] and skin [32,33,34], etc. The delivery and absorption of insoluble drugs is always a key problem in the drug delivery system, and electrospinning is an effective solution [35,36,37]. Electrospinning has important applications in drug delivery systems because of its products with high porosity, high encapsulation efficiency and large specific surface area [38,39]. In a drug delivery system, release of one or more drugs can be controlled by transforming the structure of nanomaterials or using polymer carriers with different hydrophilic properties. In the terms of hydrophilicity of the matrix materials, the electrospun nanomaterials prepared from hydrophilic materials can release the drugs with poor water solubility immediately [40]; nanomaterials produced from hydrophobic materials can release the drugs in a sustained manner [41]; nanocomposites created from a combination of both hydrophilic and hydrophobic materials can release the drugs in a biphasic way [42,43]. Complicated nanostructures can further enrich the strategies for tailoring the drug controlled release profiles, for example, single-fluid electrospun or electrosprayed monolithic composites are often utilized for providing the typical immediate or sustained release profiles [44,45]. Whereas, core-shell [42,46] or Janus [47] nanostructures are frequently explored to offer biphasic drug release behaviors and tri-chamber nanostructures are able to provide even complex drug controlled release profiles such as three-phase pulsatile release [41,48].

According to their chemical composition and function, nanostructures prepared by electrospinning can be designed into two independent parts, which have raised wide concern over the years [43,49,50]. There are two different two-chamber structures as known: the one possesses the inside and outside (core-sheath structure) [27,48,49], and the other has the neighbor relation (side-by-side structure, known as Janus) [42,43,50].

Janus is the ancient Roman “double faced God”, also known as the double-sided particle. Double-sided particles refer to a class of anisotropic particles with different physical or chemical properties in two different directions, and these two parts are separated in space that each part can be designed, respectively, [51,52]. Moreover, the two parts are closely connected at the interface, which will produce an interface effect and bring new functions to the materials [52]. The name of Janus particles was first proposed by De Gennes at the Nobel Prize awarding conference in 1991, when he named a particle with a polar side and a non-polar side [53]. In the last decades, Janus technology has developed rapidly and many synthesis methods have been explored and proposed, and diversified Janus materials have been reported [51,52,54,55]. Side-by-side electrospinning process is a remarkably used method in the preparation of Janus nanofibers. In this process, hydrodynamics, electrodynamics and rheology were involved in those complicated interactions. In the side-by-side electrospinning process, a significant is controlling the movement of two fluids synchronously under electrostatic field, which start from spinneret and end with the collector. At present, one study demonstrates the feasibility in producing Janus nanocomposites with a home-made eccentric spinneret. In addition, a new hydrophilic Janus structural nanocomposite, which is successfully prepared, designed for transmembrane penetration and speedy dissolution of the poorly water-soluble herbal helicid [56].

Janus particles and Janus nanofibers have been widely reported, but the study of Janus beads-on-a-string nanostructures (also known as “beaded nanofibers”) is still largely ignored. It is generally considered that beads-on-a-string are undesirable “by-products” that must be removed to collect smooth nanofibers and uniform particles [57]. Therefore, the beads-on-a-string structures are always being avoided by optimizing the experimental conditions and parameters of the electrospinning process. In recent years, with the increasing advantages of micro-nano structures, some researchers have noticed unique features of beads-on-a-string and discovered that drug release could be adjusted by controlling the size of particles [58,59]. According to the research findings, beads-on-a-string can be prepared by uniaxial and coaxial electrospinning [60,61,62,63,64,65,66], and particle diameter can be adjusted through changing polymer solution concentration and drug-loaded [59].

In this work, Janus beads-on-a-string was prepared by a side-by-side electrospinning process with a home-made eccentric spinneret. One side of the Janus beads-on-a-string used a hydrophilic polymer (polyvinylpyrrolidone K90, PVP K90) as the nanofiber forming polymer and loaded methylene blue (MB)as a model drug, while the other side used a hydrophobic polymer (ethyl cellulose, EC) as particle forming polymer and carries ketoprofen (KET) as a model drug. Two drug release behaviors were different, because the different polymer matrices and structures in Janus nanocomposite. In addition, the relationship between structures and properties of material could be established, which would instruct to control the release of double drugs. 

## 2. Experimental

### 2.1. Materials

Ethyl cellulose (EC, 6 mPa·s to 9 mPa·s) was bought from Shanghai Aladdin Chemistry Ltd. (Shanghai, China). Polyvinylpyrrolidone K90 (PVP K90, ${\bar{M}}_{W}$ = 1,300,000) was supplied from Shanghai Sigma-Aldrich Corp. (Shanghai, China). Ketoprofen (KET) was provided from Wuhan Fortuna Chemical Ltd. (Wuhan, China). Methylene blue (MB), analytical grade dichloromethane (DCM), N, N-dimethylformamide (DMF) and methanol were supplied by Shanghai Sinopharm Chemical Reagent Ltd. (Shanghai, China). 

### 2.2. Electrospinning

The working solutions, consisting of 0.4/0.5/0.6/0.8 g PVP K90 and 50 mg MB in 10 mL methanol, were prepared for the round side of side-by-side electrospinning. The other solutions were composed of 0.7 g EC and 0.2 g KET in a mixed solvent with a volume ratio of DMF and DCM (1:1), which were used for the crescent side of electrospinning. 

In this study, four Janus structures (Janus beads-on-a-string and Janus nanofibers) were manufactured. The specific parameters of preparing side-by-side electrospun nanostructures are shown in Table 1. 

Working fluids were poured into 10 mL plastic syringe, and syringes were settled on two pumps, respectively, and led to connect a home-made eccentric side-by-side spinneret. Two working fluids were driven simultaneously through KSD100 and KSD200 syringe pumps (Cole-Parmer, Vernon Hills, IL, USA). The electrostatic power is obtained from a high-voltage power supply (ZGF 60 kV/2 mA, Wuhan Huatian Corp., Wuhan, China). The fluid flow rate of the crescent side was 0.8 mL·h^−1^, and the round side was 1.0 mL·h^−1^. For optimization, the applied voltage between the eccentric spinneret and the grounded collector was fixed at 12.0 kV, and the collection distance was 16 cm. Relative humidity and Working environmental temperature were 55 ± 5% and 25 ± 5 °C, respectively, through all electrospinning processes.

### 2.3. Characterization

#### 2.3.1. Morphology

The prepared Janus nanostructures were covered with gold for 120 s under a nitrogen atmosphere to give them electrical conductivity. The morphology of Janus nanostructures was evaluated using scanning electron microscopy (SEM, FEI Quanta 450 FEG, FEI Corporation, Hillsboro, OR, USA). The sizes of 100 nanofibers and microparticles were measured from scanning electron microscopy images, and the average diameters of nanofibers and particles were reckoned employing Image J (NIH, Bethesda, MD, USA).

#### 2.3.2. Physical Status of Components and Compatibility

Using Bruker-AXS diffractometer (Karlsruhu, Germany), XRD analysis of the simple, including raw KET/PVP/EC/MB powders and Janus nanostructures, were analyzed over the 2θ angle range of 10° to 60°. The voltage applied was 40 kV and the electric current was 30 mA.

The raw powders and Janus nanostructures were evaluated by FTIR Spectrometer (PerkinElmer, Billerica, MA, USA). The spectrum was recorded with a resolution of 2 cm^−1^ and within the scope of 500 cm^−1^ to 4000 cm^−1^. 

#### 2.3.3. In Vitro Dissolution Tests

Referring to the Pharmacopoeia of Chinese (2020 Ed.), in vitro drug dissolution tests were implemented successfully. Here, 44.4 mg Janus nanostructures were immersed in 200 mL phosphate buffer saline (PBS, pH 7.0, 0.1 M) at 37 ± 1 °C, and that is a method using constant temperature shaker device (set the speed to 50 rpm). At predetermined time points, 4.0 mL aliquot of the samples was taken out of the solution and replaced with fresh PBS to hold the volume constant. The amounts of KET and MB released were analyzed at λ_max_ = 260 nm and 664 nm using a UV-vis spectrophotometer (UV-2102PC, Unico Instrument Co., Ltd., Shanghai, China). 

The accumulative percentage of KET and MB released from the Janus nanostructures was figured according to Equation (1) [42]:

(1) $$P\left(\%\right)=\frac{c_{n}\times V_{0}+\sum_{i=1}^{n-1}c_{i}\times V}{Q_{0}}\times 100$$

where $V_{0}$ is the volume of medium dissolved (200 mL), $Q_{0}$ is the total amount of drugs in the nanostructures (mg), $c_{n}$ is the drug concentration concluded at No. n (mg·L^−1^), and $c_{i}$ is the drug concentration determined at No. i (mg·L^−1^), V is the volume of taken out the sample (4 mL). The cumulative percentages as average values were plotted as a function of time (*t*, *h*), and the experiments were implemented 6 times.

After the dissolution tests, the residues of F2 in the bulk dissolution media were separated through a TL-5.0W Table Centrifuge at 5000× *g* (Shanghai Centrifugal Machinery Research Institute Co., Ltd., Shanghai, China), and then were dried in a DHG-101-OA Electric Oven (60 °C with blowing, Zhengzhou Ansheng Scientific Instrument Co., Ltd., Zhengzhou, China) to constant weight for SEM assessments. 

## 3. Results and Discussion

### 3.1. Side-By-Side Electrospinning

**Figure 1:**
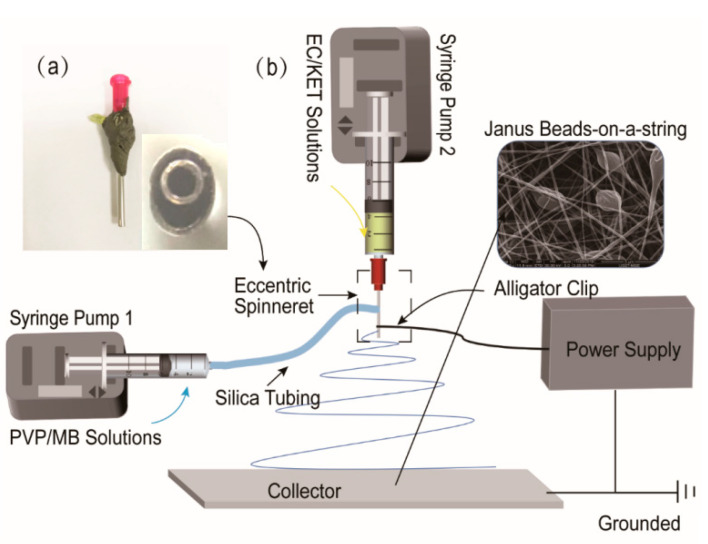
The implementation process of side-by-side electrospinning: (**a**) an image of a home-made eccentric spinneret; (**b**) schematic drawing of the home-made electrospinning system.

A diagram of the side-by-side electrospinning process are exhibited in Figure 1b, this set includes two syringe pumps, a high-voltage power supply, a collector and a home-made eccentric spinneret. Similar to a coaxial electrospinning system, side-by-side electrospinning treats two working fluids synchronously for manufacturing Janus beads-on-a-string structures, which are composed of nanofibers and microparticles. Janus beads-on-a-string make two diverse sides bound together and directly contact with the surroundings [67]. According to the latest reports, beads-on-a-string fibers were manufactured by single-fluid electrospinning and coaxial electrospinning [68,69]. In addition, the fabrication of Janus beads-on-a-string by using a parallel spinneret are rarely seen. Thus, a home-made eccentric needle was fabricated, and side-by-side electrospinning process was carried out to prepare the Janus beads-on-a-string nanostructures. A digital image of the home-made eccentric spinneret, presented in Figure 1a, nested one metallic capillary into the other one and deflected on one side. The advantage of the eccentric spinneret is that it does not separate the two working fluids under the function of high-voltage electrostatic.

**Figure 2:**
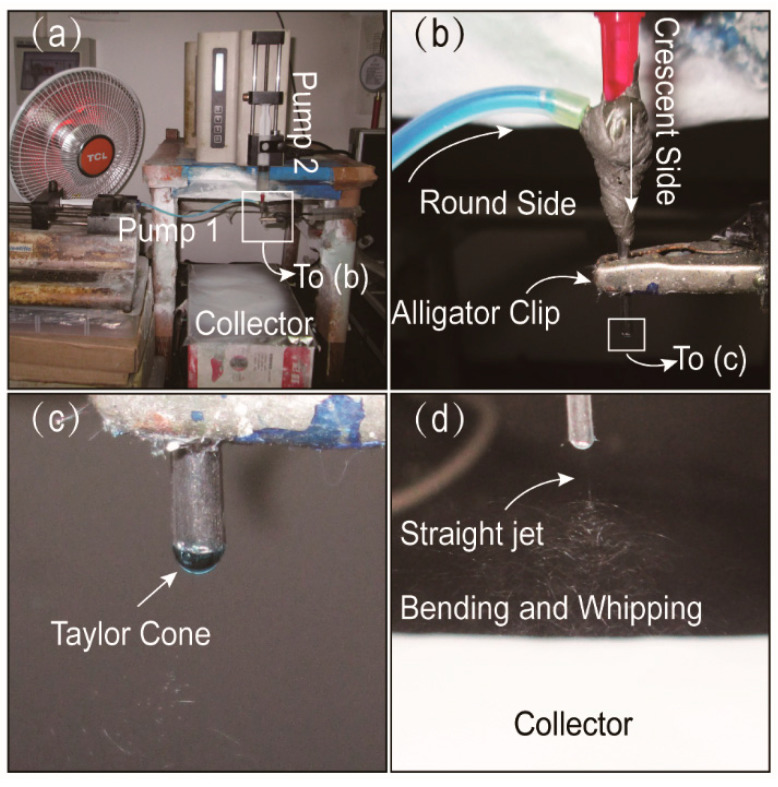
Observations of the side-by-side electrospinning: (**a**) the home-made side-by-side electrospinning system; (**b**) the connection of spinneret with two working fluids and power supply; (**c**) compound Taylor cone (12 kV); (**d**) typical fluid jet trajectory (12 kV).

A digital image of the home-made side-by-side electrospinning system appeares in Figure 2a. Two syringe pumps were used for driving two different working fluids independently. The syringe containing the PVP-MB solution fixed to the pump was directly linked to the round side of the spinneret through a silicone tube. The other side of EC-KET solution loaded into another syringe pump was connected to the crescent side. High-voltage electrostatic can be transmitted into all fluids utilizing an alligator clip. Figure 2b shows the process of connecting the spinneret with fluids and the high-voltage.

Under the pre-optimized working parameters (voltage of 12 kV, the flow rate of 0.8/1.0 mL·h^−1^ for EC/PVP side), all fluids could work continuously and stably in side-by-side electrospinning. A typical Taylor cone was shown in Figure 2c. Additionally, Figure 2d depicts that two working fluids were concurrently ejected from the Taylor cone and moved through the straight jet, followed by bending and whipping. The solvent of working solutions evaporated, and the polymers solidified and dried on the collector to form fiber mats. The typical phenomenon indicated that two sides had better compatibility and not separate under the high-voltage electrostatic field. By the way, solvent selection is a key issue for implementing a successful single-fluid electrospinning process in terms of solubilities of the solutes, spinnability of the blending solution and also the exhaustion in the solid nanofibers for applications such as drug delivery and tissue engineering [70,71,72,73,74]. For the double-fluid side-by-side process, the selections of solvents for both sides should be paid more attention to ensure the two working fluids have good compatibility. 

### 3.2. Morphology

**Figure 3:**
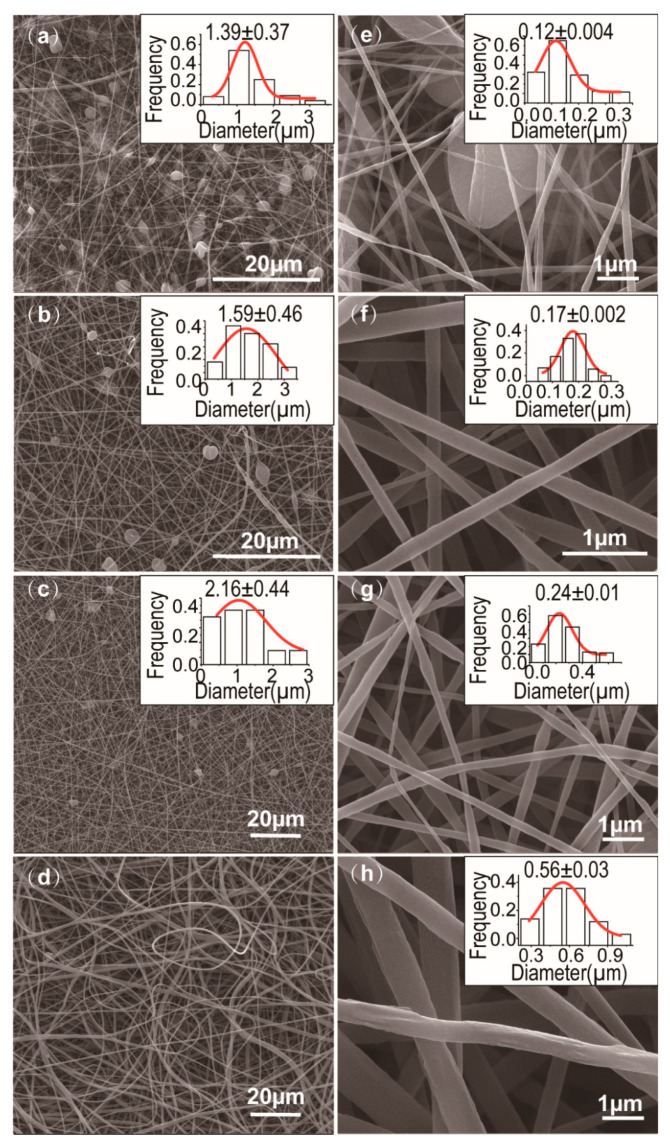
SEM images, average diameters and size distributions of the electrospun nanocomposites: (**a**,**e**) F1; (**b**,**f**) F2; (**c**,**g**) F3; (**d**,**h**) F4; (**a**–**c**) particle diameter distributions of the Janus nanostructures; (**e**–**h**) nanofiber diameter distributions of the Janus nanostructures.

The SEM images of surface morphology of Janus nanostructures (F1~F4) and their average diameters and size distributions are displayed in Figure 3. Few distinct drug crystal particles occurred on the surface of the Janus nanostructures, demonstrating that the polymers had better compatibility with MB and KET. F1~F3 are Janus beads-on-a-string and F4 is Janus nanofibers. As a general phenomenon, the beads-on-a-string nanofibers have continually appeared in electrospinning. 

As expected, the SEM images of the Janus nanostructures (includes beads-on-a-string and nanofibers) were obtained by changing PVP concentration. When PVP concentration was 4% (*w*/*v*), the nanostructures have distinct beads that include spindle-shaped and concave-shaped beads (Figure 3a). When PVP concentration was 5% or 6% (*w*/*v*), the nanostructures have been mostly concave beads and the latter has fewer microparticles than the former (Figure 3b,c). However, Janus beads-on-a-string was no longer produced and Janus nanofibers were formed as the PVP concentration reached 8% (*w*/*v*) (Figure 3d). From Figure 3a to Figure 3d, with the augment of polymer concentration, we can be preliminarily found that the numbers of microparticles have reduced in Janus nanostructures. Compared with the single electrospinning of PVP concentration of 4% (*w*/*v*), the 5% (*w*/*v*) concentration of nanofibers performs well linear morphology clear of spindles or beads phenomenon. As a result, the minimum spinnable PVP concentration (5% (*w*/*v*)) can be combined with EC solution to prepare Janus beads-on-a-string, and most of the microparticles are EC particles. When the concentration of PVP increased to 8% (*w*/*v*), the spinnable PVP can drive the unspinnable EC solution to form Janus nanofibers.

The Janus nanocomposites F1, F2, F3 (beads-on-a-string) and F4 (nanofibers) prepared through side-by-side electrospinning have average nanofiber diameters of 0.12 ± 0.004 µm (Figure 3e), 0.17 ± 0.002 µm (Figure 3f), 0.24 ± 0.01 µm (Figure 3g) and 0.56 ± 0.03 µm (Figure 3h), respectively. Three kinds of Janus beads-on-s-string F1-F3 have severally average microparticle diameters of 1.39 ± 0.37 µm (Figure 3a), 1.59 ± 0.46 (Figure 3b) µm and 2.16 ± 0.44 (Figure 3c) µm. Based on the foregoing information, the following result can be drawn: with the increase of polymer concentration on one side, the number of microparticle is decreased and the average diameters of nanofiber and microparticle are increased (except for 8% (*w*/*v*) PVP concentration). By comparison to the smooth surfaces of Janus nanofibers F4 (or core/sheath nanofibers), beads-on-a-string has ultrafine nanofibers to release drugs rapidly. Moreover, it also has larger microparticles as a storehouse to conduct sustained release.

### 3.3. Physical Form of Components and Compatibility

**Figure 4:**
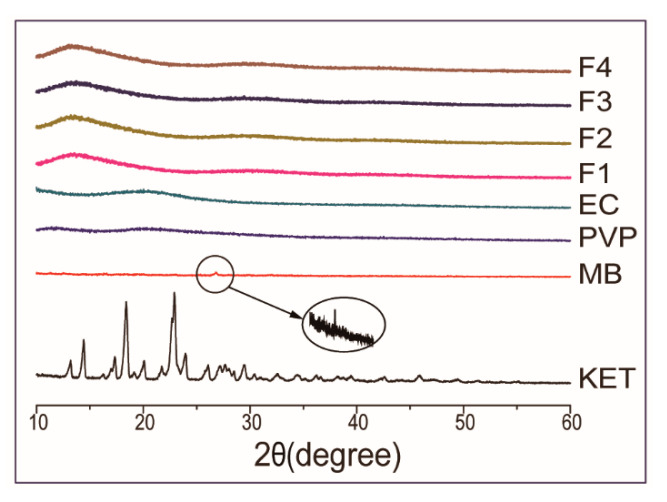
XRD patterns of raw powders and Janus nanostructures.

The XRD patterns of raw powders (PVP, EC, KET, MB) and the Janus nanostructures (F1, F2, F3, F4) are shown in Figure 4. The sharp peaks at around 26.8° of MB powders showed their crystalline characteristic. Many sharp reflections of raw KET powders were found in the XRD pattern, proving that the raw drug was a crystalline material. On the contrary, the XRD patterns of PVP/EC had two weak and wide diffraction peaks, indicating that they are an amorphous state. In the XRD patterns of F1, F2, F3 and F4, the distinctive reflections of MB and KET were not discovered. Four Janus nanostructures prepared by side-by-side electrospinning process had similar patterns with the patterns of PVP and EC, which present wide diffraction speaks and there were no sharp peaks (crystalline characteristic). Under the action of the high-voltage electrostatic field, the crystalline drugs (KET and MB) were dispersed in the matrix materials (PVP and EC) in the form of small molecules and existed in the electrospun Janus nanostructures with the amorphous state. The observation indicated that MB and KET as crystalline materials were completely transformed into an amorphous state by the process. The results of XRD confirmed that the Janus beads-on-a-string and Janus nanofibers were amorphous solid dispersions, which could be utilized to figure out the dissolution of the problem with poor water-solubility drugs [35].

**Figure 5:**
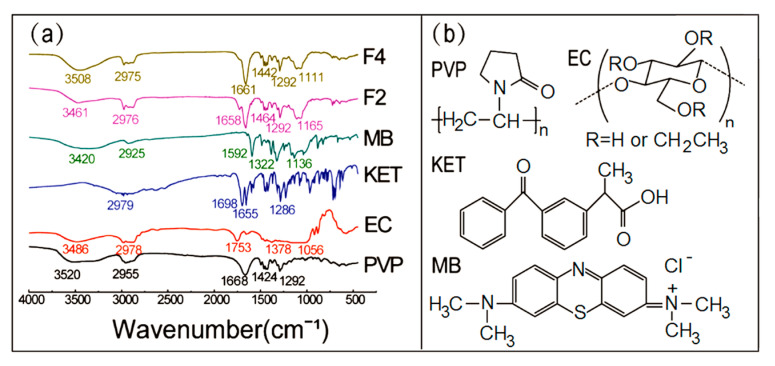
Compatibility investigation: (**a**) ATR-FTIR spectra of the components (MB, KET, EC, PVP) and their side-by-side electrospun nanostructures F2 (Janus beads-on-a-string), F4 (Janus nanofibers); (**b**) molecular structures of PVP, EC, KET, MB.

The compatibility between the drugs and polymers was investigated by FTIR analysis, which is necessary for preparing steady and high-quality Janus nanostructures. The secondary interactions, such as hydrophobic interaction, electrostatic attraction and hydrogen bonding enhanced the compatibility among the components [68]. The ATR-FTIR spectrum of raw powders and Janus nanostructures are shown in Figure 5a. The spectrum of PVP showed a typical broad water absorption peak at 3520 cm^−1^. Then, the characteristic peak at 1668 cm^−1^ was aroused by C=O stretching vibration and the peak at 1424 cm^−1^ was produced by -CH bending vibration, and the peak at 1292 cm^−1^ was raised by C-N stretching vibration. With regards to EC, the characteristic peaks at 1378 cm^−1^ for -CH bending, 3486 cm^−1^ for -OH stretching vibration and 1056 cm^−1^ for C-O stretching are shown in its spectrum. In the spectrum of KET, 1698 cm^−1^ and 1655 cm^−1^ for stretching vibrations of the carbonyl group and ketone group were observed. The typical peak at 1592 cm^−1^ for benzene ring skeletal stretching vibration, 1322 cm^−1^ and 1136 cm^−1^ for C-N and C-S stretching vibrations were shown in the spectrum of MB. The peaks at 2955 cm^−1^, 2978 cm^−1^, 2979 cm^−1^, 2925 cm^−1^ were the stretching vibrations of C-H in the spectrum of PVP, EC, KET and MB, respectively. Whereas, the peak at 1698 cm^−1^ of KET was vanished in the spectrum of F2 and F4, proving that hydrogen bonds were formed between the proton donor of EC and the receptors of KET. As was known to all, methylene blue had strong adsorption for polymers and no concern about their compatibility. The results of ATR-FTIR demonstrated that the components had well compatibility and stability.

### 3.4. In Vitro Drug Release Profiles

KET and MB have maximum UV absorbance peaks at 260 nm and 664 nm, respectively. According to the predetermined calibration curve $A_{1}=0.0508C_{1}+0.0757\left(R_{1}=0.9996\right)$ and $A_{2}=0.0696C_{2}+0.04219\left(R_{2}=0.9953\right)$, where $C_{1}$ and $C_{2}$ are the concentration of KET and MB (µg·mL^−1^) and $A_{1}$ and $A_{2}$ are the sample absorbance at 260 nm and 664 nm, the sum release of KET and MB from the Janus beads-on-a-string can be determined by UV-vis.

**Figure 6:**
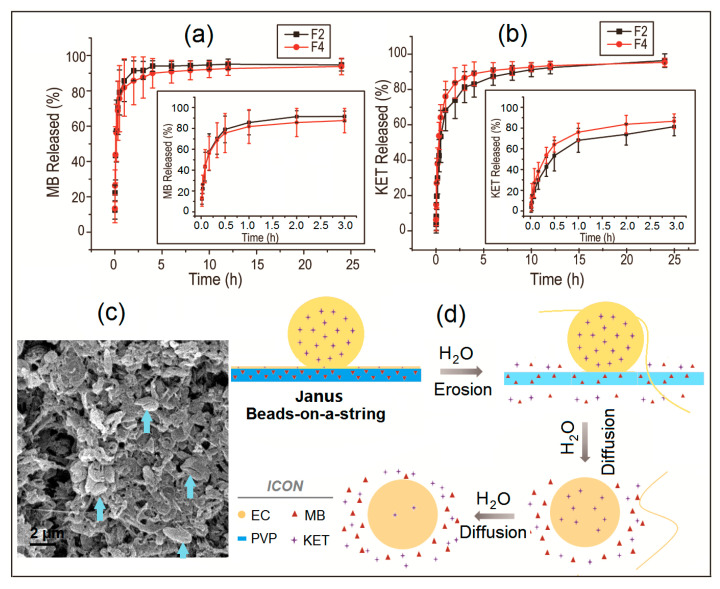
In vitro drug release profiles and mechanisms: (**a**) MB; (**b**) KET; (**c**) An SEM image of the residue EC particles after in vitro dissolution tests; (**d**) A diagram about drug release mechanisms.

The in vitro drug controlled release profiles of the Janus beads-on-a-string F2 and Janus nanofibers F4 are concluded and compared in Table 2 and Figure 6. Consistent with the concept design, F2 and F4 released near 80% of the loaded MB within 30 min (Figure 6a,b), suggesting an immediate release of MB from the PVP sides in the Janus structures. Many reports demonstrated that PVP nanofibers loaded with the drugs dissolve immediately in several seconds and release the drugs of nanofibers completely [48,56,75,76]. Slightly different from those studies, the other side of Janus nanostructures is hydrophobic polymeric beads of EC. Additionally, the sectional encapsulation of the linear PVP sides in the EC sides should slow down the fast release of MB. After the in vitro dissolution tests, the residues from F2 were observed using SEM, whose images are given in Figure 6c. The beads with lines in them (indicated by arrows) give a hint that (1) the linear sides in the original beads-on-a-string structures were the water soluble polymer PVP and MB; (2) the bead sides were the insoluble EC and the loaded KET; and (3) linear PVP sides were sectionally encapsulated by EC beads before dissolution and created lines on the EC beads in the SEM images. 

Meanwhile, as shown in the inset of Figure 6a and Table 2, MB release from Janus beads-on-a-string F2 was faster than Janus nanofibers F4 slightly. The reason for this phenomenon can be concluded that the nanofiber sides of Janus beads-on-a-string products have smaller diameter than Janus nanofibers. The release of MB did not reach 100% quickly because it was a dye that could be absorbed to water-insoluble EC particles easily. No matter Janus beads-on-a-string products F2 or Janus nanofibers F4, the MB release mechanism is the erosion mechanism, in which the drug is released by the dissolution of polymer carrier [40], as indicated in Figure 6d. 

In contrast, KET release from the EC sides is controlled by the typical diffusion mechanism because of the insolubility of EC and the homogeneous distribution of KET molecules in the EC matrices. Shown in Figure 6b and Table 2, F2 provided a better sustained release profile than F4 distinctly (an estimated 3.00 and 1.50 h was needed for F2 and F4 to release 80% of the loaded KET, Table 2). The reason is that the microparticles of Janus beads-on-a-string structures formed by EC can be regarded as a “drug repository”. Those microparticles of F2 have larger diameters than the linear EC sides of F4, and thus provide a longer diffusion distance for the penetration of water and KET molecules, by which a slower drug sustained release could be ensured.

The KET release profiles from Janus beads-on-a-string F2 and Janus nanofibers F4 were analyzed employing the Peppas Equations (2) and (3) [69]:

(2) $$Q=\frac{M_{t}}{M_{\infty }}=kt^{n}$$

(3) $$log\frac{M_{t}}{M_{\infty }}=nlog\left(t\right)+\log\left(k\right)$$

where n is the release index that figures the drug release mechanism, k is the kinetic parameter, $M_{t}$ is the sum of drug released at time t, t is the release time, $M_{\infty }$ is the sum of the drug released and Q is the percentage of the drug released.

The equation of regression for F2 are $Q_{MB1}=64.5t^{0.24}\left(R=0.7436\right)$ and $Q_{KET1}=44.2t^{0.40}\left(R=0.8436\right)$; the regressed equation for F4 are $Q_{MB2}=63.8t^{0.22}\left(R=0.7551\right)$ and $Q_{KET2}=52.8t^{0.33}\left(R=0.8146\right)$. These results show that the release index *n* is smaller than the critical value of 0.45, which indicates that drugs controlled release from side-by-side electrospinning products can be controlled through a typical mechanism of Fickian diffusion regardless of Janus nanofibers or Janus beads-on-a-string products. These results suggested that the structure of the product had no significant effect on the drugs diffusion mechanism.

The above study demonstrated that drug release could be controlled through adjusted the polymer concentration, and alter the nanomaterials structure. The Janus beads-on-a-string F2, prepared by the process, is more advantageous than the Janus nanofibers F4 in the aspects of controlled release profiles of double drugs and the potential combined therapy. The ultrafine hydrophilic nanofibers of the Janus beads-on-a-string released the first drug (MB) more rapidly than Janus nanofibers, while the larger hydrophobic microparticles of the Janus beads-on-a-string released the second drug (KET) more slowly than Janus nanofibers. Compared with Janus nanofibers, it provided more accurate control over the release of double drugs.

A schematic diagram of drugs release mechanisms is given in Figure 6d. When dissolved in the phosphate buffer saline, the PVP side nanofibers of the Janus beads-on-a-string structures will dissolve at a high rate of speed and release all the drugs that were contained through a typical erosion mechanism. The rest of EC particles side provides the sustained release loaded drugs through a typical diffusion mechanism. Due to EC is insoluble in water, there are still some residual drugs within the microparticles that cannot be completely released after the testing time of 24 h. The Janus structure is tunable and can be adjusted by changing the polymer composition on both sides of the structure to manipulate the release behaviors of drugs.

## 4. Summary

Janus beads-on-a-string composite structures composed of PVP-MB//EC-KET were successfully achieved by a side-by-side electrospinning process. SEM images showed the fibers and particles of the Janus beads-on-a-string clearly. Through changing the polymer concentration, Janus beads-on-a-string with different particle distribution and diameter could be obtained. XRD patterns proved that all the components were present in the Janus nanostructures with amorphous states. ATR-FTIR spectrum testified that the compatibility and stability of the components were very well due to hydrogen bonding. In vitro drug dissolution tests demonstrated that the Janus beads-on-a-string could provide typical double drugs controlled release profiles, which had the faster immediate release and slower sustained release than Janus nanofibers. The drugs released from the Janus beads-on-a-string were controlled through a representative mechanism of Fickian diffusion. The current work showed a new preparation method of the Janus beads-on-a-string structures using side-by-side electrospinning, which also provided a fresh idea for double drugs controlled release and the combined medication.

## Figures and Tables

**Table 1:** Specific parameters for the side-by-side electrospun nanostructures.

No.	Process	PVP Side		EC Side		Morphology
		Flow Rate ^1^	PVP ^2^	Flow Rate ^1^	EC ^3^	
F1	Side-by-side Electrospinning	1.0	0.4	0.8	0.7	Janus beads-on-a-string
F2		1.0	0.5	0.8	0.7	
F3		1.0	0.6	0.8	0.7	
F4		1.0	0.8	0.8	0.7	Janus nanofibers

^1^ Flow rate: mL·h^−1^. ^2^ Concentration: g·10 mL^−1^; PVP side consists of 50 mg MB in methanol. ^3^ Concentration: g·10 mL^−1^; EC side consists of 0.2 g KET in a mixed solvent with a volume ratio of DMF and DCM (1:1).

**Table 2:** A comparison of the drug release behaviors from F2 and F4.

Tested Samples	MB Released (%)		KET Released (%)		Time for KET Release (h) ^a^	
	0.5 h	24 h	0.5 h	2 4 h	30%	80%
F2 Janus Beads-on-a-string	79.10 ± 12.78	94.72 ± 3.48	53.37 ± 14.72	96.34 ± 3.82	0.17	3.00
F4 Janus nanofibers	75.57 ± 18.75	93.78 ± 4.73	64.16 ± 7.28	95.34 ± 2.40	0.13	1.50

^a^ The value indicates that the time needed for reaching a certain accumulative release of KET and is achieved by Interpolation method.

## Data Availability

The data presented in this study are available on request from the corresponding author. The data are not publicly available because that they are a portion of Ding Li’s thesis for Master degree.
